# Outpatient treatment of decompensated heart failure: A systematic review and study level meta‐analysis

**DOI:** 10.1002/ehf2.14841

**Published:** 2024-07-16

**Authors:** Jameela Bahar, Amna Rahman, Grace W.Y. Wong, Rajiv Sankaranarayanan, Fozia Z. Ahmed, Rebecca Taylor, Ahmet Fuat, Iain Squire, John G.F. Cleland, Gregory Y.H. Lip, James H.P. Gamble, Sundas Masudi, Prince Josiah S. Joseph, Kenneth Y.K. Wong

**Affiliations:** ^1^ Department of Cardiology, Lancashire Cardiac Centre Blackpool UK; ^2^ Lancashire Cardiac Centre Patient Public Involvement Group, Lancashire Cardiac Centre Blackpool Teaching Hospitals NHS Foundation Trust Blackpool UK; ^3^ Liverpool University Hospitals NHS Foundation Trust Liverpool UK; ^4^ Liverpool Centre for Cardiovascular Science University of Liverpool Liverpool UK; ^5^ Keele University, Keele Cardiovascular Research Group Keele UK; ^6^ Department of Cardiology Manchester University Hospitals NHS Foundation Trust Manchester UK; ^7^ Durham University UK; ^8^ NIHR Cardiovascular Research Centre Glenfield Hospital Leicester UK; ^9^ British Heart Foundation Centre of Research Excellence, School of Cardiovascular and Metabolic Health University of Glasgow Glasgow UK; ^10^ Liverpool Centre for Cardiovascular Science at University of Liverpool Liverpool John Moores University and Liverpool Heart and Chest Hospital Liverpool UK; ^11^ Department of Clinical Medicine Aalborg University Aalborg Denmark; ^12^ Oxford Heart Centre John Radcliffe Hospital Oxford UK

**Keywords:** outpatient IV diuretics, acute decompensated heart failure, systematic review, meta‐analysis

## Abstract

Patients with acutely decompensated heart failure (ADHF) are usually admitted to hospital for management. There is growing interest in delivering intravenous (IV) diuretic therapy at home, in the community or at hospital day‐care units; the safety and effectiveness of outpatient‐based management (OPM) for ADHF has not been established. We conducted a systematic literature review and meta‐analysis to investigate the short‐term safety and effectiveness of OPM compared with inpatient management (IPM) of ADHF. Pre‐specified endpoints were 30 day mortality and 30 day hospitalization. The meta‐analysis was conducted using RevMan 5.4 software. Twenty‐nine studies of OPM were identified, including 7683 patients. Only five studies directly compared OPM (*n* = 1303) with IPM (*n* = 2047), including three observational studies, and two randomized controlled trials (RCTs). The other 24 studies only stated OPM outcomes. For the five studies comparing IPM versus OPM, patients were generally aged >75 years and of similar age for each strategy, with a similar proportion of men (56%). In a study‐level, aggregate analysis, 30 day all‐cause mortality was 9.3% (121/1303) for OPM, compared with 15.6% (320/2047) for IPM [OR 0.29 (95% CI 0.09, 0.93) *P* = 0.04]. Four studies reported 30 day all‐cause hospitalization; 22.0% for IPM versus 16.8% for OPM [OR 0.73 (95% CI 0.61, 0.89), *P* = 0.001]. In the two RCTs, we found no difference in 30 day mortality or hospitalization. In observational studies, OPM of ADHF is associated with lower 30 day hospitalization and lower 30 day mortality; such differences were not observed in two small, single‐centre RCTs. A substantial, multicentre RCT is required to confirm the safety and effectiveness of OPM for ADHF.

## Introduction

Acute heart failure (AHF) is the primary reason for about 100 000 hospitalizations each year in the United Kingdom,^1^ with a median length of stay of 5 days for those patients not seen by specialists and 8 days for those seen by heart failure (HF) specialists or in cardiology wards. Inpatient mortality was 10%.^2^ For some patients with AHF, alternatives to hospitalization may be appropriate. Indeed, some centres have initiated outpatient intravenous (IV) diuretic programmes (furosemide lounges/day‐case/ambulatory care unit in hospital, in the community or at home).^3^, ^4^ Recently, a systematic review of 11 observational studies reported that outpatient management (OPM) with intravenous (IV) diuretics was safe and associated with lower mortality rates than inpatient management (IPM). However, only patients who were considered to have a good short‐term prognosis received OPM, potentially biasing the results.^5^ The review highlighted the need to consider other outcomes, including quality of life and re‐hospitalization rates, and concluded that a sufficiently powered randomized controlled trial (RCT) was required to demonstrate the safety and utility of OPM. We recently conducted a pilot RCT including 24 patients and became aware of another small RCT. Accordingly, we decided to update the existing systematic review.

### Objectives

This systematic literature review and meta‐analysis investigates the effectiveness of OPM of AHF using IV and subcutaneous diuretics compared with IPM (considered current standard of care). The primary outcomes of interest were 30 day mortality and 30 day re‐hospitalization.

## Methods

### Literature search strategy

Relevant publications were identified by online search engines (Ovid and Scopus) and reference lists from recent systematic reviews and abstracts/conference proceedings.^5^, ^6^ Our database search terms incorporated: Heart failure, Diuretics, Outpatient, Intravenous and Subcutaneous (‘heart failure’ AND ‘diuretics’ AND ‘outpatient’ AND (‘intravenous’ OR ‘subcutaneous’).

Inclusion criteria consisted of patients with acute/worsening HF and publications written in English. There was no limit applied to the duration of follow‐up or the sample size, but case reports (*n* = 1) were excluded. Reviews, summaries and book chapters were also excluded.

### Study selection and data extraction

Two first‐authors (A. R and J. B.) independently screened the titles and abstracts of all articles found by searches to identify potentially eligible publications, for which full texts were subsequently obtained and reviewed. Where uncertainty existed, the senior author (K. Y. K. W.) was consulted. Data were then extracted (A. R. and J. B.), including baseline clinical characteristics, selection criteria (inclusion and exclusion), study measurements, treatment plans and outcomes [all‐cause and HF hospitalizations, mortality, other adverse events, and New York Heart Association (NYHA) class]. Observational studies were assessed for risk of bias by the reviewing authors (A. R. and J. B.) using the six factor Quality in Prognosis Studies (QUIPS) tool.^7^ RCTs were assessed using the Risk of Bias 2 (RoB2) tool.^8^ Pre‐specified endpoints were 30 day mortality and 30 day hospitalization.

### Meta‐analysis methodology

#### Data synthesis

Data from eligible trials were entered into the RevMan 5.4 software package.^9^ Where applicable, for dichotomous data (30 day mortality and 30 day rehospitalization), the odds ratio (OR) and 95% confidence intervals (CIs) were calculated. The *I* ^2^ statistic was used to quantify heterogeneity. The results from the trials were pooled using a fixed effects model if the *I* ^2^ statistic (heterogeneity) were sufficiently low. If the *χ* ^2^ statistic was *P* < 0.10, a random effect model was used to allow generalization of the results.^10^

In addition, we performed an aggregate analysis for all the OPM studies and the five studies comparing OPM with IPM (*Figure* S1): weighted mean age and % male patients were calculated for both OPM and IPM.

## Results

**Figure 1:**
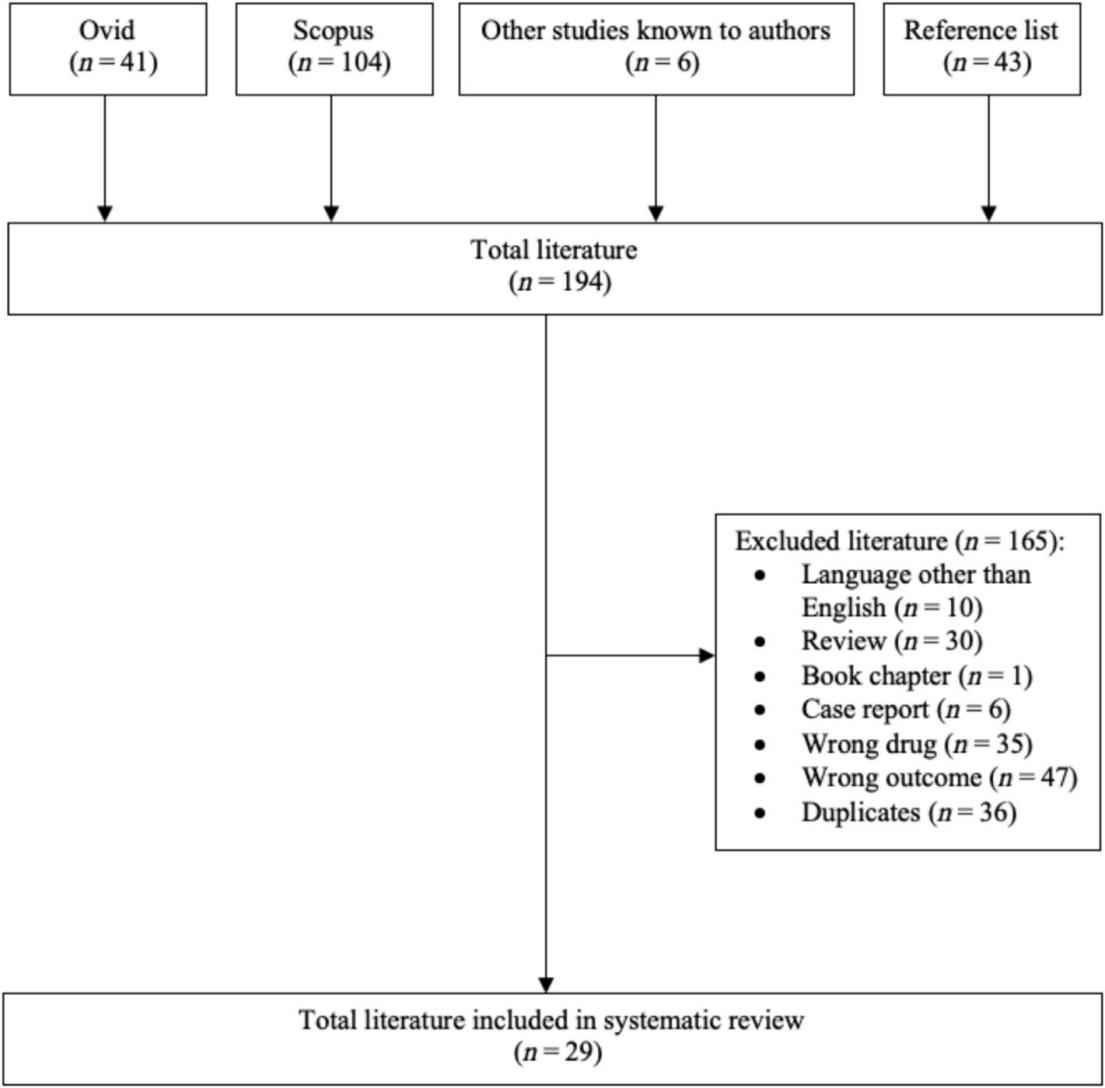
Flowchart demonstrating the literature selection and inclusion process.

Our systematic literature search identified 194 published studies, of which 165 publications were excluded. Thus, 29 publications met the inclusion criteria, studying OPM for AHF using parenteral diuretics (*Figure* 1). The studies included 9730 patients, 7683 who had OPM and 2047 who had IPM.

Of the 29 studies, 5 compared OPM with IPM for the pre‐specified endpoints of 30 day mortality and hospitalization. This included three observational studies, two of which were conference proceedings,^11^, ^12^, ^13^ and two small single‐centre RCTs with fewer than 100 patients.^14^, ^15^

There was significant heterogeneity amongst studies in baseline clinical characteristics (*Table* 1 and S1A). With the exception of one observational study,^16^ all studies enrolled more men than women. Patients who received OPM were younger (62 years) and less likely to be women (37%) than those who received IPM (77 years and 44%, respectively). The diuretic regime and duration of follow up in each study are summarized in Table S1B. Few studies provided information about changes in plasma concentrations of natriuretic peptides (6/29) and NYHA class before and after treatment (4/29) (Table S2). Table S2 summarizes the endpoints reported in each study.

**Table 1:** Study Design and Patient Demographics of the studies included in the meta‐analysis

Author	Study type	Inclusion	Exclusion	No. patients	Age	Sex (male)	Aetiology	Mean LVEF (%)	NYHA class	Renal function
Hamo et al (2021)^15^	Single centre RCT (OPM vs. IPM) (US)	Patients >18 years old with known heart failure pathophysiology and clinical features	Significant comorbid condition	94	63.8 ± 12.9	56.4%	NA	33.5 ± 19.3	12.9% II 26.9% III 58.1% IV	BUN 29.8 ± 14 mmol/L. Serum creatinine 1.27 ± 0.42 mg/dL
Ahmed et al (2021)^11^	Single‐centre observation study (OPM vs. IPM) (UK)	Adults in the community not responding to increasing doses of oral diuretic treatment who are willing and able to complete IV diuretic decompensation treatment on an outpatient basis	Patients with hemodynamic instability and signs of shock. Or secondary causes of decompensation	154 (IP = 75 and OP = 79)	IP—72 (36–94) OP—77 (49–93)	IP—60% (*n* = 45) OP—57% (*n* = 45)	IPs with HFrEF 74.7% (*n* = 56) OPs with HFrEF 46.8% (*n* = 37)	NA	NA	NA
Salmon et al (2021)^12^	Single‐centre observation study (OPM vs. IPM) (UK)	NA	NA	2901	IP—77.1 ± 10.2 OP—74.2 ± 9.1	IP—55.4% OP—57.1%	NA	NA	NA	NA
Wong et al (2021)^14^	Single‐centre pilot RCT (OPM vs. IPM) (UK)	NA	NA	IP (*n* = 11) OP (*n* = 13) *N* = 24	IP 81.8 (10.4) OP 70 (16.0)	IP 36.4% OP 76.9%	IHD: IP 18.2% and OP 7.7%	NA	IP: III—11 (100%) OP: II—2 (15.4%) III—8 (61.5%) IV—3 (23.1%)	Urea: IP—11.35 (4.4) mmol/L. OP—10.2 (5.1) mmol/L. Creatinine: IP—119.5 (37) umol/L. OP—113.7 (48) umol/L
Thomas et al (2019)^13^	Single‐centre observational study (OPM vs. IPM) (UK)	Exacerbation of heart failure needing diuretics	NA	206 patients (208 admissions) IP—36 (17.3%), not admitted—172 (82.7%)	IP—78.3 ± 15.2. Not admitted—78.8 ± 12.0.	IP—18 (50%). not admitted—77 (44.8%)	NA	NA	NA	NA

Abbreviations: IPM, inpatient management; LVEF, left ventricular ejection fraction; NYHA, New York Heart Association; OPM, outpatient‐based management; RCT, randomized controlled trial.

### Thirty day mortality and 30 day hospitalization

Based on an analysis of aggregated data from the publications included in this systematic review which included mortality data (*n* = 13), overall, 30 day mortality was 8.9% for OPM (180 deaths from 2026 patients) (*Table* 2).

**Table 2:** The study population of studies included in statistics for 30 day mortality, 30 day hospitalization and 30 day all‐cause hospitalization for OPM and IPM

		30 day mortality	30 day HF hospitalization	30 day all‐cause hospitalization
OPM	Number of studies with relevant data	12	10	11
	Total population	2026	5162	6268
	Affected population	180	668	986
	%	8.9%	13%	16%
IPM	Number of studies with relevant data	5	3	4
	Total population	2047	122	1972
	Affected population	320	19	443
	%	16%	16%	22%

Only studies that reported 30 day outcomes were included in this analysis (*Table* 2).

Abbreviations: IPM, inpatient management; OPM, outpatient‐based management.

From the five publications which compared OPM with IPM, 121/1303 (9.3%) who received OPM died within 30 days, compared with 320/2047 (15.6%) for IPM (*Figure*s 2 and S2A,B). For these five studies, the IPM cohort were on average only 2 years older (mean age 77 vs. 75 in the OPM studies) with a similar proportion of male patients (55.5 vs. 55.6%). The three observational studies showed lower mortality for OPM compared with IPM (9.5% vs.16.0%), but the two RCTs did not (0 vs. 0 in Hamo *et al*.'s trial of 94 patients in the United States,^15^ and 7.7% vs. 9.1% in Wong *et al*.'s trial in the United Kingdom).^14^

**Figure 2:**
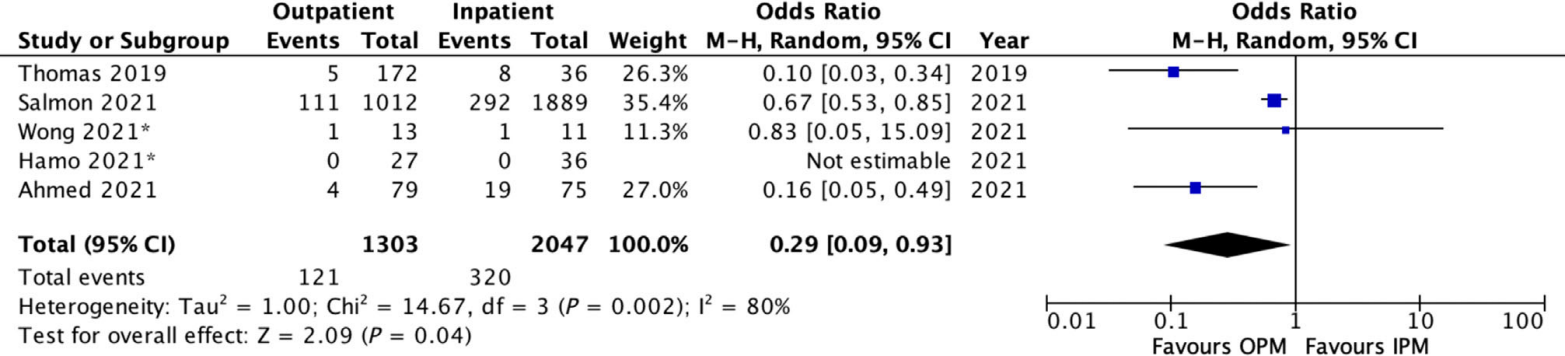
A forest plot showing 30 day mortality of the studies directly comparing outpatient management versus inpatient management of acute decompensated heart failure using intravenous diuretics.

**Figure 3:**
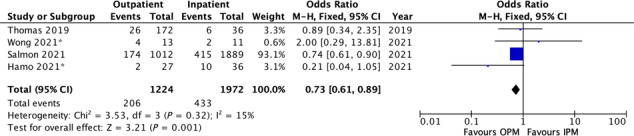
A forest plot showing 30 day hospitalization of the studies directly comparing outpatient management versus inpatient management of acute decompensated heart failure using intravenous diuretics.

Using data from the studies that assessed these endpoints, all‐cause and HF 30 day rehospitalization were 16% (986/6268) and 13.0% (668/5162) for OPM.^12^, ^13^, ^14^, ^15^, ^17^, ^18^, ^19^, ^20^, ^21^, ^22^, ^23^ Four publications comparing OPM‐ versus IPM‐reported 30 day hospitalization.^12^, ^13^, ^14^, ^15^ Of the OPM cases, 206/1224 (16.8%) were hospitalized within 30 days versus 433/1972 (22%) IPM cases. OPM was associated with lower 30 day hospitalization risk [OR 0.73 (95% CI 0.61, 0.89), *P* = 0.001] (*Figure* 3 and S3A,B).

Five publications reporting a composite outcome of 30 day death or hospitalization,^11^, ^15^ showed that in 149 patients receiving OPM, 33 died or were admitted within 30 days (22%).

The risk of bias was deemed lower in clinical trials (Table S3A) compared with the observation studies (Table S3B). We found better outcomes for patients receiving OPM for AHF in observation studies, but this was not replicated in two small single‐centre RCTs.

### Randomized trials

In the first UK single‐centre feasibility RCT of 24 patients, patients treated by OPM had significantly more days alive out of hospital (within 30 days of randomization), with no excess mortality observed (1/11 vs. 1/13).^14^ No excess mortality was observed up to 60 days of follow‐up (2/11 vs. 2/13).^14^ In this RCT, Adult State Hope scores were increased more with OPM within 30 days but dropped to lower levels than IPM by 60 days possibly because numerically more OPM patients were admitted within 60 days (6/13 patients randomized to OPM vs. 2/11 inpatients).^24^ More outpatients had increased total well‐being scores by 60 days (*P* = 0.04) and OPM was associated with estimated mean cost savings of £2,658 (95% central range 460–4857) per patient.^14^, ^24^, ^25^

A second small, single‐centre trial (OUTLAST)^15^ in the United States demonstrated that IV furosemide was more effective than IV saline at reducing 30 day rehospitalization for HF (3.7% vs. 23%, P = 0.037). No significant differences in clinical outcomes were reported between the groups.

In addition, we also found lower HF hospitalization for OPM was observed in the RCT by Hamo *et al*.^15^ [16.7% (6/36) vs. 3.7% (1/27)]. In the pilot RCT in the United Kingdom, Wong *et al*.^14^ found numerically higher HF hospitalization in the OPM group [9.1% (1/11) vs. 15.4% (2/13)] (IPM vs. OPM). According to the detailed OUTLAST study protocol (https://doi.org/10.1371/journal.pone.0253014.s007—document), the ‘standard of care arm will be admitted or discharged from the emergency room based on the discretion of the physicians involved. If admitted, the subject will be treated in the usual manner.’ Thus, it is possible that at least a proportion of patients randomized to standard care (IPM) in OUTLAST were discharged from the emergency room, suggesting that they may be less sick than Wong *et al*.'s cohort. Similarly, it should be noted that the higher 30 day all‐cause hospitalization in patients randomized to OPM in the small trial (Wong *et al*.) is not replicated in the aggregate analysis. So when compared with IPM, 30 day all‐cause hospitalization and HF hospitalization is lower whether we only examine the studies that compare IN versus OUT (*Figure* 3), or all the studies that examine OPM (*Table* 2).

## Discussion

The key finding of our study‐level aggregate analysis is that the outpatient management for acute decompensated HF was associated with lower 30 day mortality compared with standard inpatient care [9.3% vs. 15.6% (5 studies)]. However, in our analysis lower mortality for OPM compared with IPM was seen only in observational studies [9.5% vs. 16.0% (3 studies)]. In contrast, there was no significant difference between the OPM and IPM 30 day mortality between the two small single‐centre trials [0 vs. 0 in Hamo *et al*.'s trial of 94 patients in the United States,^15^ and 1/13 (7.7%) vs. 1/11 (9.1%) in Wong *et al*.'s trial in the United Kingdom].^14^ This finding could indicate a degree of selection bias in the observational studies of OPM although it is likely that the two small trials do not have sufficient statistical power to detect mortality. Further, the inclusion criteria for OPM services are likely designed in such a way to be biased in favour of selecting patients at low risk of mortality, complication and rehospitalization.

In the small RCT of 24 patients in the United Kingdom, 4/13 (30.8%) of OPM were hospitalized within 30 days versus 2/11 (18.2%) of inpatients.^14^ In contrast, the US RCT reported lower 30 day hospitalization in the OPM cohort (7.4% vs. 27.8%),^15^ which is in keeping with lower 30 day hospitalization in favour of OPM reported in the two observational studies (16.9% vs. 21.9%).^12^, ^13^ However, according to the detailed study protocol of the US RCT, the standard of care arm would be admitted or discharged from the emergency room based on the discretion of the physicians involved. If admitted, the subject would be treated in the usual manner, but there were no details on what proportion of patients in the standard of care group were admitted to hospital receiving IV diuretics. It should also be noted that comparing these studies is difficult with the different healthcare systems.^15^

### Strengths and limitations

In Wierda *et al*.'s review,^5^ the authors excluded research in emergency departments or observation units and studies where only the abstract was available. We have attempted to include abstracts/conference proceedings as well as searched reference list from reviews.^5^, ^6^ Nevertheless, as with all systematic reviews, this is still prone to potential publication bias.

Moreover, one must be cautious interpreting aggregate analyses that included both RCTs and observational studies, especially given the greater influence of Salmon *et al*.'s observation study as indicated by the weightings in *Figure*s 2 and 3. Although Salmon *et al*. carries a big weighting in the 30 day mortality data, it is evident that the smaller observation studies outcomes also appear statistically significantly better for OPM. In Wong *et al*.'s small trial, there was numerically lower 30 day mortality (but not statistically significant). However, Wong *et al*.'s small trial suggested there may be a signal of concern with numerically higher 30 day hospitalization in patients randomized to OPM. Nevertheless, all the observation studies and Hamo's small trial suggest OPM is associated with reduced 30 day hospitalization. Thus, meta‐analysing all the trials and observation studies may dampen the signal of concern.

It is possible that observational studies showed better outcomes in OPM groups as patients with fewer comorbidities were selected. Two small RCTs do not have enough patients to draw any meaningful conclusions from. This is why we urgently need a large randomized trial to inform future HF international guidelines. Heterogeneity may render the pooled effects unreliable. Even amongst the observational studies comparing IPM with OPM, there was significant heterogeneity (*P* = 0.0007) (*Figure* S2A). We have therefore used a random effect model to potentially improve generalization of the results. In this study level meta‐analysis, we have attempted to compare age and gender proportion in the studies that directly compared OPM with standard inpatient care, to assess possible selection bias. However, there are other sources of heterogeneity which we are unable to fully examine in this study‐level meta‐analysis. It is also important to note that many studies failed to report consistent patient BNP levels, which is known to be strongly associated with mortality.

### A trend of expansion of OPM despite limited evidence from RCTs

Wierda's systematic review in 2020 reported that OPM is safe with some observational data suggesting that OPM was associated with relatively low mortality rates; however, one major limitation was the absence of comparison with inpatients.^5^

In the United States, of over 1.1 million unique HF visits, across >11 000 hospitals and outpatient clinics, 1% received outpatient IV diuretics in 2015.^26^ This has doubled compared with 2006 before the ‘Hospital readmissions reduction programme’ in 2012. Nearly 19% of hospitals administered outpatient IV diuretics.^26^ There was a decrease in hospitalization to less than 30%, and a slight increase in observation units to around 2%, whereas the emergency department discharges stayed around 4.5% and standard clinic visits accounted for >60%.^26^ This US trend may be driven by reimbursement rules, which do not provide institutional reimbursement for HF patients who are readmitted within 30 days.

In the United Kingdom, OPM service also appeared to have gained popularity fairly quickly according to two surveys.^3^, ^4^ An estimated 25 485 patients per year [median 600, interquartile range (IQR 295–800) per site] received inpatient care for ADHF while 2731 per year patients [median 50 (7–100) per site] received OPM for AHF, representing 9.7% of total ADHF population in the 2021 survey.^4^ The 2021 survey also confirms there is uncertainty/equipoise amongst the HF community in the United Kingdom about whether to develop this service.

Ambulatory emergency care is an increasingly prevalent model of acute care. Although the potential to manage acute HF in ambulatory care is recognized,^27^ there are no standardized guidelines for how to achieve this.

### Future research

We hope to perform a patient‐level meta‐analysis of studies comparing IPM versus OPM and apply artificial intelligence/machine learning algorithms to produce a risk score to predict likelihood of success of OPM. This patient‐level meta‐analysis will also enable us to fully investigate sources of heterogeneity which might include age, gender, renal function, frailty, BNP/NTproBNP, ejection fraction, sodium and haemoglobin. This study will provide vital data for the next steps towards precision medicine in this field. Furthermore, improvements in quality of life can be measured with the Kansas City Cardiomyopathy Questionnaire (KCCQ) to identify any differences between OPM and IPM.

The small pilot trial in Blackpool suggested that OPM is effective, safe and cost effective and is a strategy favoured both by patients and carers.^14^, ^24^, ^25^ Importantly, patients randomized to OPM appeared to enjoy an improvement of their mental well‐being. OPM was estimated to save the NHS in excess of £2600 per patient compared with IPM.^25^ Although patients randomized to OPM appeared to have increased levels of hope initially, by 60 days follow‐up, their levels of hope dropped possibly because there were numerically (albeit not statistically significantly) higher number of readmissions by 60 days. In our present systematic review and aggregate analysis of the 29 studies, HF hospitalization was lower in the OPM group within 30 days (13% vs. 16%). The US pilot trial also reported lower 30 day HF hospitalization for OPM 3.7% (1/27) versus 17% (6/36).^15^ The planned large multicentre trial will help to resolve the uncertainties HF services have regarding whether to develop outpatient based IV diuretic treatment for AHF.

### Patient public involvement

The Lancashire Cardiac Centre Patient Public Involvement (PPI) group consists of patients, carers and members of the public diversified in age, gender and ethnicity. This meta‐analysis is considered to be very important because patients have indicated that it is more meaningful to feel well and be out of hospital rather than simply staying alive.

Wong *et al*.'s small pilot trial^14^, ^24^, ^25^ suggests that patients who receive OPM enjoy more days alive outside of hospital but signals a potentially higher risk of 30 day hospitalization. Our present meta‐analysis shows that in selected patients receiving OPM in observational studies, 30 day hospitalization is in fact lower than patients receiving standard IPM care. We contend that although unpublished abstracts may not yet have been peer reviewed as rigorously, it is a strength to include them in the meta‐analysis to minimize the risk of publication bias.

The PPI group unanimously agree that there is a need to perform a large multicentre RCT to test the safety and effectiveness of OPM.

## Conclusions

Outpatient IV diuretics for acute/worsening HF appears safe and effective in observation studies and two small single‐centre RCTs although one of the small trials suggests possible increase in 30 day hospitalization in patients randomized to OPM. A large prospective multicentre RCT is required to determine safety, clinical effectiveness and cost effectiveness, in order to inform international HF guidelines.

### Sources of Funding

This work was supported by British Heart Foundation Clinical Research Collaborative to K.Y.K.W. and the University of Liverpool INSPIRE to J. B. and A. R. (joint first authors). We are also grateful for the funding of the open access publication article processing fees by the Manchester University NHS Foundation Trust.

## Supporting information


**Figure S1.** Identifying and comparing OPM vs IPM papers and statistics.

**Figure S2.** Forrest Plots showing 30‐day mortality data papers included in the meta‐analysis.
**Figure S2A.** 30‐day mortality data of the observational papers included in the meta‐analysis.
**Figure S2B.** 30‐day mortality data of the randomized control trial papers included in the meta‐analysis.
**Figure S3.** Forrest Plots showing 30‐day hospitalization data papers included in the meta‐analysis.
**Figure S3A.** 30‐day hospitalization data of the observational papers included in the meta‐analysis.
**Figure S3B.** 30‐day hospitalization data of the randomized control trial papers included in the meta‐analysis.
**Table S1.** A summary of the papers identified in the literature review.
**Table S1A.** Selection criteria and patient characteristics of papers identified in the literature review.
**Table S1B.** An overview of study protocols and treatment regimes of papers identified in the literature review.
**Table S2.** Endpoints and clinical study outcomes of papers identified in the literature review.
**Table S3.** Quality assessments using objective risk of bias tools.
**Table S3A.** A Quality assessment of Randomized control trials reviewed using the RoB2 tool.
**Table S3B.** A Quality assessment of the Observational studies reviewed using QUIP's tool.

## References

[1] British Heart Foundation, Heart and Circulatory Disease Statistics 2022 . [Internet]. 2022. Available from: https://www.bhf.org.uk/‐/media/files/research/heart‐statistics/bhf‐statistics‐compendium‐2022.pdf?rev=79c10677e14141ee886970ac9808f1db&hash=79A256DC5330081D89E5D5124E1F60EC [last accessed on 29th November 2022].

[2] National Institute for Cardiovascular Outcomes Research . National Heart Failure Audit 2022 summary report (2020/2021 data) [Internet]. 2020. Available from: https://www.nicor.org.uk/wp‐content/uploads/2022/06/NHFA‐DOC‐2022‐FINAL.pdf [last accessed on 21st September 2022].

[3] Mohee K , Wong K . Rapid response to “how to prescribe loop diuretics in oedema”. BMJ 2019;364:l359. doi:10.1136/bmj.l359 30792231

[4] Abdullah A , Wong SYS , Jones R , Wong KYK . Development of outpatient based acute heart failure care calls for development of clinical psychology service for whole‐person care provision. Br J Cardiol 2022;29:141–144. doi:10.1136/bmj.l359 PMC10270302 37332271

[5] Wierda E , Dickhoff C , Handoko ML , Oosterom L , Kok WE , de Rover Y , *et al*. Outpatient treatment of worsening heart failure with intravenous and subcutaneous diuretics: a systematic review of the literature. ESC Heart Fail 2020;7:892–902. doi:10.1002/ehf2.12677 32159279 PMC7261522

[6] Lee C , Beleznai T , Hassan S , Rawat A , Douglas H , Kanagala P , *et al*. Ambulatory management of acute decompensation in heart failure. Br J Hosp Med 2019;80:40–45. doi:10.12968/hmed.2019.80.1.40 30592667

[7] The Cochrane Collaboration Prognosis Methods Group, Review Tools . 2018. Available from: http://methods.cochrane.org/sites/methods.cochrane.org.prognosis/files/uploads/QUIPS%20tool.pdf [last accessed on 20th April 2022].

[8] Sterne JAC , Savović J , Page MJ , Elbers RG , Blencowe NS , Boutron I , *et al*. RoB 2: a revised tool for assessing risk of bias in randomized trials. BMJ 2019;366:l4898. doi:10.1136/bmj.l4898 31462531

[9] Review Manager (RevMan) [Computer program] . Version 5.4, The Cochrane Collaboration, 2020. Available from: https://training.cochrane.org/online‐learning/core‐software‐cochrane‐reviews/revman [last accessed on 20th April 2022].

[10] Brown OI , Allgar V , Wong KY‐K . Coffee reduces death risk after acute myocardial infarction: a meta‐analysis. Coron Artery Dis 2016;27:566–572. doi:10.1097/MCA.0000000000000397 27315099

[11] Ahmed FZ , Taylor JK , John AV , Khan MA , Zaidi AM , Mamas MA , *et al*. Ambulatory intravenous furosemide for decompensated heart failure: safe, feasible, and effective. ESC Heart Fail 2021;8:3906–3916. doi:10.1002/ehf2.13368 34382749 PMC8497198

[12] Salmon T , Essa H , Brousas S , Whybrow‐huppatz I , Balu A , Jackson C , *et al*. Long term outcomes of ambulatory outpatient management of acute decompensated heart failure in a heart failure specialist nurse delivered specialist unit versus inpatient management. Circulation 2021;144:A14004. doi:10.1111/jnc.16117

[13] Thomas K , Martin J , Watkinson O , Wicks E , Jackson H , Bone R , *et al*. Ambulatory management of patients with acute heart failure: setting up a multi‐disciplinary service. Presented in British Society of Heart Failure Meeting 2019.

[14] Wong KYK , Hughes DA , Debski M , Latt N , Assaf O , Abdelrahman A , *et al*. Effectiveness of out‐patient based acute heart failure care: a pilot randomised controlled trial. Acta Cardiol. 2023;78:828–837. doi:10.1080/00015385.2023.2197834 37694719

[15] Hamo CE , Abdelmoneim SS , Han SY , Chandy E , Muntean C , Khan SA , *et al*. OUTpatient intravenous LASix trial in reducing hospitalization for acute decompensated heart failure (OUTLAST). PLoS ONE 2021;16:e0253014. doi:10.1371/journal.pone.0253014 34170908 PMC8232441

[16] Fort A , Luiso D , Blázquez‐Bermejo Z , Calvo‐Fernández A , Martínez‐Medina F , García‐Ribas C , *et al*. Ambulatory intravenous treatment of decompensated heart failure: an effective, safe and cost‐effective approach. REC: CardioClinics 2021;56:7–13.

[17] Banerjee P , Tanner G , Williams L . Intravenous diuretic day‐care treatment for patients with heart failure. Clin Med (Lond) 2012;12:133–136. doi:10.7861/clinmedicine.12-2-133 22586787 PMC4954097

[18] Brightpurpose . Evaluation of IV diuretics pilot for BHF. Final report. [Internet] . 2014. Available from: https://www.bhf.org.uk/‐/media/files/hcps/bh0101‐00‐‐‐ivd‐final‐report.pdf [last accessed on 20th April 2022].

[19] Buckley LF , Seoane‐Vazquez E , Cheng JW , Aldemerdash A , Cooper IM , Matta L , *et al*. Comparison of ambulatory, high‐dose, intravenous diuretic therapy to standard hospitalization and diuretic therapy for treatment of acute decompensated heart failure. Am J Cardiol 2016;118:1350–1355. doi:10.1016/j.amjcard.2016.07.068 27772698

[20] Buckley LF , Stevenson LW , Cooper IM , Knowles DM , Matta L , Molway DW , *et al*. Ambulatory treatment of worsening heart failure with intravenous loop diuretics: a four‐year experience. J Card Fail 2020;26:798–799. doi:10.1016/j.cardfail.2019.10.015 31704197

[21] Zuzarte P , Kostiw K , Maciukiewicz M , Figueira ML , Costa‐Vitali A . Outpatient disease management program for heart failure: a multidisciplinary approach with an ambulatory intravenous diuretic therapy. Insuficiencia Cardiaca 2018;13:2–9. doi:10.1016/j.recesp.2013.06.010

[22] Ioannou A , Browne T , Jordan S , Metaxa S , Mandal AKJ , Missouris CG . Diuretic lounge and the impact on hospital admissions for treatment of decompensated heart failure. QJM 2020;113:651–656. doi:10.1093/qjmed/hcaa114 32251503

[23] Alghalayini KW . Effect of diuretic infusion clinic in preventing hospitalization for patients with decompensating heart failure. SAGE Open Med 2020;8:2050312120940094. doi:10.1177/2050312120940094 32670579 PMC7339079

[24] Wong K , Hughes DA , Debski M , Latt N , Assaf O , Abdelrahman A , *et al*. Does outpatient based IV diuretic treatment for acute heart failure give patients hope? Heart 2021;107:A116–A117.

[25] Wong K , Assaf O , Latt N , Debski M , Abdelrahman A , Taylor R , *et al*. Is outpatient based acute heart failure treatment cost‐effective? An analysis based on a pilot prospective trial. Heart Jul 2020;106(Suppl 2):A73–A74.

[26] Greene SJ , Wilson LE , Abbasi SA , Yusuf AA , Hammill BG . Outpatient intravenous diuretic therapy for heart failure in the United States. J Am Coll Cardiol 2019;73:1101–1103. 30846106 10.1016/j.jacc.2018.12.034 PMC7085417

[27] British Association of Ambulatory Emergency Care . Directory of ambulatory emergency care for adults [Internet]. 2016. Available from: https://www.ambulatoryemergencycare.org.uk/uploads/files/1/BAAEC/AEC%20Directory%202016%205th%20edition.pdf.pdf [Last accessed on 20th April 2022].

