# Supplementary material for: Outpatient treatment of decompensated heart failure: A systematic review and study level meta‐analysis
Source: ESC Heart Fail. 2024 Jul 16;12(2):761–9. doi: 10.1002/ehf2.14841 (PMC11911626; doi:10.1002/ehf2.14841)
Supplement: Supplementary file 2 — Figure S2. Forrest Plots showing 30‐day mortality data papers included in the meta‐analysis. Figure S2A. 30‐day mortality data of the observational papers included in the meta‐analysis. Figure S2B. 30‐day mortality data of the randomized control trial papers included in the meta‐analysis. Figure S3. Forrest Plots showing 30‐day hospitalization data papers included in the meta‐analysis. Figure S3A. 30‐day hospitalization data of the observational papers included in the meta‐analysis. Figure S3B. 30‐day hospitalization data of the randomized control trial papers included in the meta‐analysis. Table S1. A summary of the papers identified in the literature review. Table S1A. Selection criteria and patient characteristics of papers identified in the literature review. Table S1B. An overview of study protocols and treatment regimes of papers identified in the literature review. Table S2. Endpoints and clinical study outcomes of papers identified in the literature review. Table S3. Quality assessments using objective risk of bias tools. Table S3A. A Quality assessment of Randomized control trials reviewed using the RoB2 tool. Table S3B. A Quality assessment of the Observational studies reviewed using QUIP's tool. [file EHF2-12-761-s001.docx]

**Supplementary information (Part 1 – XL spreadsheet):**

**Suppl. Figure 1. Identifying and comparing OPM vs IPM papers and statistics**

- submitted as a separate XL file.

**Supporting Information - Part 2:**

Supplementary Figures:

Supplementary Figure 2: Forrest Plots showing 30-day mortality data papers included in the meta-analysis

Supplementary Figure 2A: 30-day mortality data of the observational papers included in the meta-analysis

Supplementary Figure 2B: 30-day mortality data of the randomized control trial papers included in the meta-analysis

Supplementary Figure 3: Forrest Plots showing 30-day hospitalization data papers included in the meta-analysis

Supplementary Figure 3A: 30-day hospitalization data of the observational papers included in the meta-analysis

Supplementary Figure 3B: 30-day hospitalization data of the randomised control trial papers included in the meta-analysis

Supplementary Tables:

Supplementary Table 1: A summary of the papers identified in the literature review

Supplementary Table 1A: Selection criteria and patient characteristics of papers identified in the literature review

Supplementary Table 1B: An overview of study protocols and treatment regimes of papers identified in the literature review

Supplementary Table 2: Endpoints and clinical study outcomes of papers identified in the literature review

Supplementary Table 3: Quality assessments using objective risk of bias tools

Supplementary Table 3A: A Quality assessment of Randomized control trials reviewed using the RoB2 tool

Supplementary Table 3B: A Quality assessment of the Observational studies reviewed using QUIP’s tool

Supplementary References

**Supplementary Figure 2: Forrest Plots showing 30-day mortality data papers included in the meta-analysis**

**Supplementary Figure 2A: 30-day mortality data of the observational papers included in the meta-analysis**


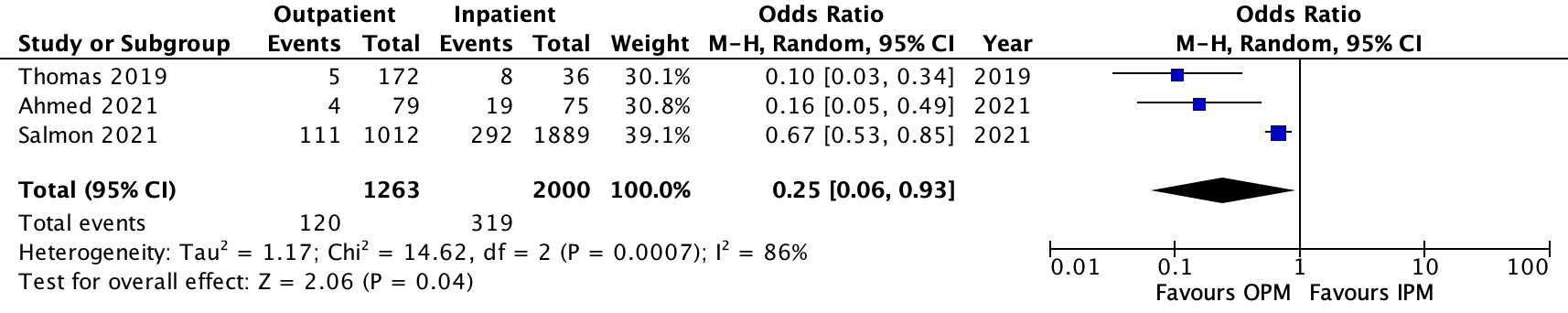


**Supplementary Figure 2B: 30-day mortality data of the randomized control trial papers included in the meta-analysis**


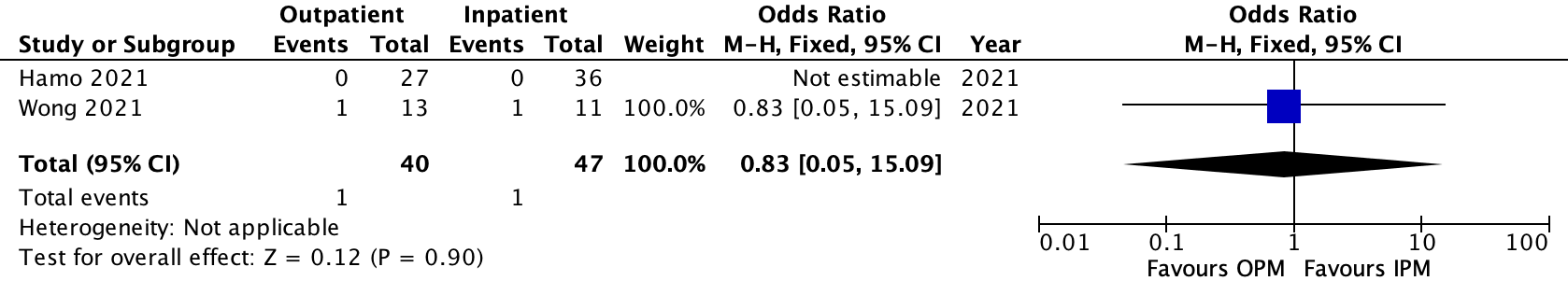


**Supplementary Figure 3: Forrest Plots showing 30-day hospitalization data papers included in the meta-analysis**

**Supplementary Figure 3A: 30-day hospitalization data of the observational papers included in the meta-analysis**


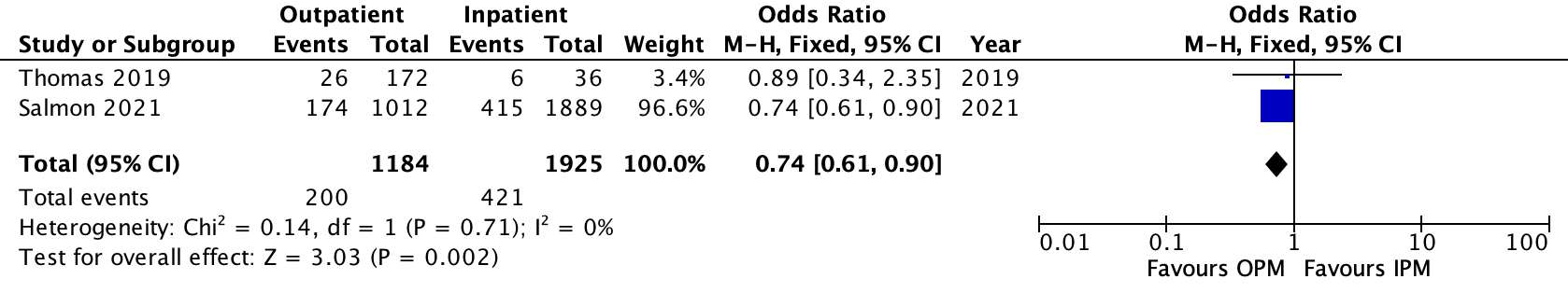


**Supplementary Figure 3B: 30-day hospitalization data of the randomised control trial papers included in the meta-analysis**


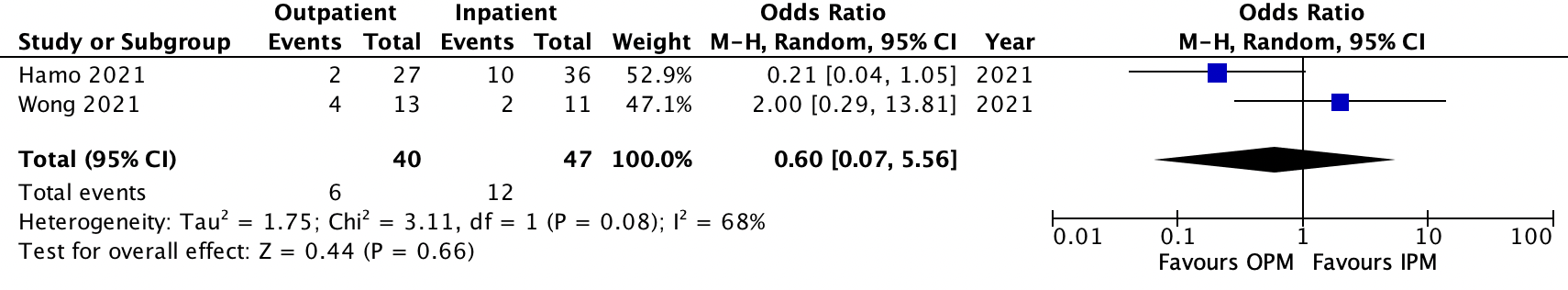


**Supplementary Tables 1: A summary of the papers identified in the literature review**

**Supplementary Table 1A: Selection criteria and patient characteristics of papers identified in the literature review**

| **Author** | **Study Type** | **Inclusion** | **Exclusion** | **No. patients** | **Age** | **Sex (male)** | **Aetiology** | **Mean LVEF (%)** | **NYHA**  **Class** | **Renal function** |
| --- | --- | --- | --- | --- | --- | --- | --- | --- | --- | --- |
| Ryder et al (2008)^28^ | Single centre observational study (OPM only) (Ireland) | Weight gain >2kg in 2 days and symptoms | NA | 107 | 71 ± 11 | 80% | 70% HFrEF, HFpEF 30% | 38 ± 15 | 8%II, 83%III, 8%IV | NA |
| Freimark et al (2009) ^29^ | Single centre observational study (OPM only) (Israel) | Congestion, recent hospitalization | UAP | 190 | 65 ± 12 | 89% | 77% Ischemic Cardiomyopathy (iCMP) | 25 ± 11 | 100% III and | Creatinine 1.7 ± 0.7 mg/ml |
| Herbert et al (2011)^30^ | Single centre observational study (OPM only) (US) | Weight gain >2.2kg and symptoms | NA | 130 | 58 ± 13 | 72% | 35% iCMP | 23 ± 10 | 2% I, 26% II, 29% III, 33% IV | eGFR 70 ml/min |
| Lazkani et al (2012)^31^ | Case series (OPM only) | Weight gain >2.2kg and symptoms | NA | 7 | NA | 57% | 57% HFrEF, HFpEF 43% | NA | 100% III | NA |
| Banerjee et al (2012)^17^ | Single centre observational study (OPM only) (UK) | Congestion | Advanced chronic kidney disease | 17 | 70 ± 6 | 71% | 94% HFrEF, 58% iCMP | NA | NA | NA |
| Schipper et al (2012)^32^ | Single centre observational study (OPM only) (US) | NA | NA | 23 | 70 | NA | EF<40% (n=12), EF>40% (n=11) | NA | NA | NA |
| Zatarain et al (2013)^33^ | Single centre observational study (OPM only) (Spain) | Decompensated heart failure (SC furosemide) | NA | 24 | 75 ± 10 | 79% | 38% iCMP | 58% LVEF, <45% | III and IV | Creatinine 1.59 ± 0.59 mg/ml |
| Evaluation of IV diuretics for the British Heart Foundation [Brightpurpose (2014)]^18^ | Multicentre Observational Centre (OPM only) (UK) | Confirmed HF, living in the community, accepted by HF Nurse Service | Palliative care cases | 96 | 75 | 76% | 55% IHD | NA | 92% III or higher | NA |
| Makadia et al (2015)^34^ | Single centre observational study (OPM only) (US) | Pulmonary congestion | NA | 106 | 68 ± 13 | 53% | 64% HFrEF (iCMP), 36% HFpEF | 39 ± 18 | 3% I, 44% II, 42% III, 11% IV | Creatinine 127 micromol/l |
| Buckley et al (2016)^19^ | Single centre observational study (OPM vs IPM) (US) | Decompensated heart failure | Hypotension, hypernatremia end-stage renal disease,  significant comorbidities, or required emergency  cardiac care. | 157 Ambula-tory Diuretic Therapy  (ADT), [n=80];  (Standard care n=77) | 70 (IQR 62-80) for ADT group | 57%  (ADT group) | 56% (55%-60%) HFpEF, 20% (20%-30%) HFrEF  (ADT group) | NA | 1/70 (1%) I, 11/70 (16%) II, 41/70 (59%) III, 17/70 (24%) IV. (ADT group) | Creatinine 1.45 (IQR 1.15-1.88)  mg/dL  (ADT group) |
| Buckley et al (2016)^35^ | Single centre observational study (OPM only) (US) | Pulmonary congestion | Significant acute comorbid condition | 60 | 70 ± 10 | 57% | 60% HFrEF, 40% HFpEF | 25% in HFrEF, 55% in HFpEF | 12% II, 58% III, 20% IV, 10% unknown | Creatinine 115 micromol/l |
| Al-Ani et al (2019)^36^ | Single centre observational study (OPM only) | Hypervolemia | NA | 19 | 65 | 57% | 27% HFrEF | NA | NA | 60% eGFR <60ml/min |
| Buckley et al (2019)^20^ | Single centre observational study (OPM only) (US) | Pulmonary congestion | Significant acute comorbid condition | 283 | 68 ± 14 | 62% | NA | 49% LVEF <40% | NA | NA |
| Gilotra et al (2018)^37^ | Single centre phase II RCT [IV vs SC] (OPM only) (US) | Worsening HF (SC Furosemide) | Chance of hospitalization | 41 | 57 ± 13 | 78% | 69%, HFrEF, 33%  HFpEF | 25% (range  15-55%) | 30% II 60% III  10% IV | eGFR 62 ± 53 ml/min |
| Sica et al (2018)^38^ | Experimental PK/PD study (OPM only) (US) | Symptomatic and symptoms of chronic fluid overload for oral diuretics. | NA | Study 1 – 10  Study 2 -17 | Study 1 – 69.9 ± 8.6  Study 2 - 68.0 ± 9.5 | Study 1 – 80%  Study 2 – 88.2% | NA | NA | Study 1 - 100% II  Study 2 – 76.5% II,  23.5% III (n=17). | Study 1 – eGFR 53.8 (49.5–58.7) ml/min/1.73m^2^  Creatinine 120.0 (102.5–131.2) mmol/l.  Study 2 - eGFR 63.4 (41-97) ml/min/1.73m^2^.Creatinine 105.5 (80.5–143.24) mmol/l. |
| Zuzarte et al (2018)^21^ | Single centre observational study (OPM only) (Canada) | Patients at high risk of hospitalization | NA | 138 | 74.6 (± 11.43) | 78 (56.5%) | Ischemic: 73, (52.9%).  Non-ischemic: 65, (47.1%). | 42% | I - 11, (7.9%).  II - 63, (45.7%).  III – 60, (43.5%).  IV - 4, (2.9%). | Infused group eGFR 45 mL/min. |
| Greene et al (2019)^26^ | Multi-centre observational study (OPM only) (US) | NA | NA | 1,144,274 unique HF care visits | NA | NA | NA | NA | NA | NA |
| St Amand et al (2020)^39^ | Single centre observational study (OPM only) (US) | Hypervolemia | NA | 27 | 78.3 ± 8.3 | 100% | HFrEF 37% | 46.1 ± 12.7 | NA | eGFR- 40.3 ± 15.7 mL/min/1.73m^2^.  Serum creatinine at discharge 2.1 ± 1.4 mmol/l. |
| Ioannou et al (2020)^22^ | Single centre observational study (OPM only) (UK) | All patients with primary diagnosis of HF meeting criteria for admission into diuretic lounge | NA | 245 | 73.1 ± 13.2 | 67.4% | N/A | LVEF: >55% n=70 (28.6%). 45–55% n=95 (38.8%). 35–44% n=39 (15.9%). <35% n=82 (33.5%) | N/A | eGFR- 49.1 ± 21.3  serum creatinine  136.0 ± 64.8 mmol/L. |
| Alghalayini et al (2020)^23^ | Single centre observational study (OPM only) (Saudi Arabia) | Decompensating patients not responding to maximal tolerated oral diuretics; signs or symptoms of heart failure. | Patients in shock; severe renal dysfunction; liver failure | 105 | NA | NA | Decompensated HF | NA | NA | NA |
| Hamo et al (2021)^15^ | Single centre RCT (OPM vs IPM) (US) | Patients > 18 years old with known heart failure pathophysiology and clinical features | Significant comorbid condition | 94 | 63.8 ± 12.9 | 56.4% | NA | 33.5 ± 19.3 | 12.9% II 26.9% III 58.1% IV | BUN 29.8 ± 14 mmol/L.  Serum creatinine 1.27 ± 0.42 mg/dL |
| Verma et al (2021)^40^ | Single centre observational study (OPM only) (US) | Clinical features of acute decompensated heart failure  no acute cardiovascular issue, no NYHA IV symptoms | NA | 27 | 72 (67-80) | 93% | 52% HFrEF | NA | 100% III | Creatinine 1.59 (1.27 - 1.82) mg/dl |
| Vaishnav et al (2021)^41^ | Single centre observational study (OPM only) (US) | Diagnosis of CA | NA | 44 | 71.3 ± 9.7 | 75% | NA | 46.0 ± 12.0 | 25% II 68% III 2% IV | Creatinine 1.2 (1.1-1.9) mg/dL  eGFR 58.5 (40.3, 76.0) mL/min/1.73 m^2^ |
| Ahmed et al (2021)^11^ | Single centre  Observation study (OPM vs IPM)  (UK) | Adults in the community not responding to increasing doses of oral diuretic treatment who are willing and able to complete IV diuretic decompensation treatment on an outpatient basis. | Patients with hemodynamic instability and signs of shock. Or secondary causes of decompensation. | 154  (IP=75 and OP=79) | IP - 72 (36-94)  OP - 77 (49-93) | IP - 60% (n=45)  OP - 57% (n=45) | IPs with HFrEF 74.7% (n=56)  OPs with HFrEF 46.8% (n=37) | NA | NA | NA |
| Salmon et al (2021)^12^ | Single centre  Observation study (OPM vs IPM)  (UK) | NA | NA | 2901 | IP - 77.1 ± 10.2  OP - 74.2 ±9.1 | IP - 55.4%  OP - 57.1% | NA | NA | NA | NA |
| Wong et al (2021)^14^ | Single Centre pilot RCT (OPM vs IPM) (UK) | NA | NA | IP (n=11)  OP (n=13)  N=24 | IP 81.8 (10.4)  OP 70 (16.0) | IP 36.4%  OP 76.9% | IHD: IP 18.2% and OP 7.7% | NA | IP:  III – 11 (100%)  OP:  II - 2 (15.4%)  III - 8 (61.5%)  IV - 3 (23.1%). | Urea: IP - 11.35 (4.4) mmol/L. OP - 10.2 (5.1) mmol/L.  Creatinine: IP - 119.5 (37) umol/L. OP - 113.7 (48) umol/L. |
| Butler et al (2021)^42^ | Single centre  observational study (OPM only)  (US) | Adults aged ≥18 to <65 years  with chronic HFrEF following an acute worsening. | Congenital heart disease,  amyloidosis, heart transplantation, or left ventricular assist device  during the baseline period (12 months prior) were excluded. | 4460 | 56.1 (8.0) | 63.7% (2839) | NA | NA | NA | Creatinine clearance 4.2 ±2.7. |
| Fort et al (2021)^16^ | Single centre observational study (OPM only) (Spain) | All consecutive episodes of HF decompensation treated with IV diuretics at the HF day hospital included. | Need for hospital admission due to presence of ‘alarm signs’ (e.g., hypotension, oxygen saturations <93%), renal replacement therapy. | 119 | 75 ± 11 | 35% (n=42) | HFpEF 56% (n=67) | 52 ± 15 | NA | (n=179)  Creatinine, 1.52 ± 1.18  mg/dL  eGFR, 52 ± 26 mL/min/1.73m^2^ |
| Thomas et al (2019)^13^ | Single centre  observational study (OPM vs IPM) (UK) | Exacerbation of heart failure needing diuretics | NA | 206 patients (208 admissions) IP - 36 (17.3%), not admitted - 172 (82.7%) | IP - 78.3 ± 15.2.  not admitted - 78.8±12.0. | IP - 18 (50%). not admitted - 77 (44.8%). | NA | NA | NA | NA |
| Shah et al (2016)^43^ | Single centre  observational study (OPM only) (UK) | NA | NA | 11 | 67.25 | NA | HFpEF - 5. HFrEF - 5. | NA | NA | NA |

**Supplementary Table 1B: An overview of study protocols and treatment regimes of papers identified in the literature review**

| **Author** | **Diuretic dosage before** | **Therapy** | **Treatment regimes** | **1 visit** | **>1 visit** | **Visit per patient** | **Length of follow-up** |
| --- | --- | --- | --- | --- | --- | --- | --- |
| Ryder et al (2008)^28^ | NA | IV Furosemide | 40-80 mg bolus | 72% | 28% | NA | NA |
| Freimark et al (2009) ^29^ | NA | IV Furosemide  ± metolazone ± inotropic medication | NA | NA | NA | NA | 12 months |
| Herbert et al (2011)^30^ | NA | IV furosemide +- metolazone | 40 mg bolus + infusion 160mg | NA | NA | 1-14 | 17 months |
| Lazkani et al (2012)^31^ | NA | IV Furosemide/ bumetanide + metolazone | Dosage not mentioned | 86% | 14% | 1-2 | 30 days |
| Banerjee et al (2012)^17^ | NA | IV Furosemide | 80-100mg bolus | 83% | 17% | 1-2 | 30 days |
| Schipper et al (2012)^32^ | NA | IV Furosemide | Bolus 80 mg (median) and infusion dose was 40 mg/hr. | NA | NA | NA | NA |
| Zatarain et al (2013)^33^ | NA | SC Furosemide | Furosemide continuously SC, mean 146 mg/day | NA | NA | NA | NA |
| Evaluation of IV diuretics for the British Heart Foundation [Brightpurpose (2014)]^18^ | NA | IV furosemide | bolus doses ranging from 40-250 mg | 100% | NA | 1-2 | NA |
| Makadia et al (2015)^34^ | Mean oral 160 mg furosemide | Furosemide ± metolazone | Continuous infusion furosemide (mean 100 mg) | NA | NA | 1-22 | 90 days |
| Buckley et al (2016)^19^ | NA | IV furosemide | mean dose 260 mg | NA | NA | NA | 30 days |
| Buckley et al (2016)^35^ | Mean oral 240 mg (80-800 mg) furosemide | IV Furosemide | Bolus furosemide and continuous infusion | 55% | 45% | NA | 60 days |
| Al-Ani et al (2019)^36^ | Mean oral 160 mg furosemide or equivalent | IV furosemide | Bolus 80mg | NA | NA | 30 visits for 19 patients | NA |
| Buckley et al (2019)^20^ | Mean 396 mg ± 375 furosemide or equivalent | IV furosemide | Bolus furosemide and continuous infusion | NA | NA | 483 visits for 283 patients | NA |
| Gilotra et al (2018)^37^ | Mean 246 mg ± 167 mg  furosemide | IV or SC Furosemide | Randomization between  fixed single bolus of  furosemide SC 80 mg and  intravenous bolus of 80-160 mg  (mean 123 ± 47 mg) | NA | NA | NA | 30 days |
| Sica et al (2018)^38^ | Maintenance diuretic use - Furosemide equivalent dose. Study 1 - 44.0 ± 12.6 mg/day  Study 2 – 40mg/day | Study 1 – Furosemide SC  Study 2 - Furosemide SC  or IV | Study 1 – 80 mg oral or SC infusion of furosemide (30 mg in the first hour followed by 12.5 mg/h for 4 h).  Study 2 - 80 mg of IV furosemide (40 mg in 2 mins followed by a second 40 mg dose 2 hours later) or 80 mg of SC. | NA | NA | NA | 7+-1 day |
| Zuzarte et al (2018)^21^ | NA | IV furosemide ± metolazone. | Bolus dose 40 - 100 mg in 10 minutes followed by a continuous IV infusion rate between 10 - 40 mg/hr. For 6-8 hours. | 100% | 100% | NA | 12 months |
| Greene et al (2019)^26^ | NA | NA | NA | NA | NA | NA | 30 days |
| St Amand et al (2020)^39^ | 96% oral loop diuretics | NA | NA | NA | NA | NA | 180 days |
| Ioannou et al (2020)^22^ | NA | Furosemide IV | NA | NA | NA | 2.3 ± 0.4 | 60 days |
| Alghalayini et al (2020)^23^ | maximum tolerated dose of oral loop diuretic | Furosemide IV | NA | 53.3% | 46.7% | NA | 30 days |
| Hamo et al (2021)^15^ | NA | Group 1 – standard care  Group 2 – IV placebo infusion  Group 3 - IV furosemide | Group 1 – standard care treatment per HF guidelines  Group 2 – 20-40ml of saline IV  Group 3 – equal or higher dose of patient’s home oral dose.  Low dose (20 mg bolus with 20 mg/hour infusion), intermediate dose (40 mg bolus with 40 mg/hour infusion) and high dose (80 mg bolus with 80 mg/hour infusion).  biweekly visits for dose adjusted IV diuretics, medication adjustment and education. | 99% | 90 patients. Beyond 30 days.  180 days hospitalization 34.4% were available. | NA | 30 days |
| Verma et al (2021)^40^ | 200mg/24hr oral furosemide | IV furosemide | median dose 180mg bolus and continuous infusion | 56% | 44% | NA | 442 days (median) |
| Vaishnav et al (2021)^41^ | Oral furosemide | IV furosemide | NA | 22.7% | 77.5% | average 4.6 visits per patient | 180 days |
| Ahmed et al (2021)^11^ | NA | IV furosemide | single daily infusion, up to a maximum of 240mg over 1 hr. duration. | NA | 100% | IP- 1.2 visits per patient  OP- 1.4 visits per patients | 12 months |
| Salmon et al (2021)^12^ | NA | IV furosemide | 80-480 mg | NA | NA | 5.1 ± 2.2 | IP=46 ± 8.1 months; OP=45.5 ± 7 months |
| Wong et al (2021)^14^ | NA | IV furosemide | NA | NA | NA | NA | Up to 60 days |
| Butler et al (2021)^42^ | NA | NA | NA | NA | NA | NA | 12 months |
| Fort et al (2021)^16^ | Oral furosemide (average dose 140mg per day) and 21% patients  Hydrochloro-thiazide. | IV furosemide,  oral hydrochlorothiazide, oral potassium per treatment day | IV furosemide dose of 20mg every 30min up to a total dose of 60mg. Mean dose was 240mg IV furosemide (80mg/treatment day),  Adjuvant treatment 25mg of oral hydrochlorothiazide and 25 mEq of oral potassium given to all patients. All baseline oral diuretics are maintained. | 18% of 192 episodes | 82% of 192 episodes | Mean duration of each episode of ambulatory HF IV treatment was 3 ± 2 days. | Up to 1 year, average 28-month follow-up |
| Thomas et al (2021)^13^ | NA | NA | NA | 46 (22%) | NA | NA | 1 year |
| Shah et al (2016)^43^ | NA | IV BD Furosemide | 80mg BD. Average duration 12.36 days. | NA | NA | NA | NA |

**Supplementary Table 2: Endpoints and clinical study outcomes of papers identified in the literature review**

| **Author** | **Primary endpoint** | **Secondary endpoint** | **HF Hospitalization** | **All-cause hospitalization** | **Mortality** | **Adverse events** | **BNP/pro**  **-BNP pre or post intervention** | **NYHA pre-post** |
| --- | --- | --- | --- | --- | --- | --- | --- | --- |
| Ryder et al (2008)^28^ | Clinical stability | NA | NA | NA | NA | None | pre-BNP 1063- post BNP 892 post | 3.0 ± 0.4-2.5 ± 0.6 |
| Freimark et al (2009) ^29^ | NA | NA | 0.6 hospitalization per patient per year (mostly HF) | 0.6 hospitalization per patient per year (mostly HF) | 29% 1 year | None | NA | 100%III/IV-26%II |
| Herbert et al (2011)^30^ | Frequency of IV diuretics use in the outpatient HF disease  management program (HFDMP), | Safety (change in symptoms, blood pressure, electrolytes, and mortal-  ity),  and identifying “unique clinical  characteristics” of cohort that uses the HFDMP. | NA | NA | NA | None | NA | NA |
| Lazkani et al (2012)^31^ | NA | NA | 0% 30 days | NA | 0% 30 days | NA | NA | NA |
| Banerjee et al (2012)^17^ | Symptoms | Re-hospitalization | NA | 18% 30 days | 0% 30 days | None | NA | Half class lower in 71%, one class lower in 24% |
| Schipper et al (2012)^32^ | NA | NA | NA | 14 | NA | Minimal change in blood pressure (mean systolic BP decrease 3 mmHg), and transient hypokalaemia (no clinical arrhythmia) | NA | NA |
| Zatarain et al (2013)^33^ | NA | NA | NA | NA | NA | 1 hypokalaemia | NA | NA |
| Evaluation of IV diuretics for the British Heart Foundation [Brightpurpose (2014)]^18^ | NA | NA | NA | NA | 0% | NA | NA | NA |
| Makadia et al (2015)^34^ | Days admission | Weight loss | NA | NA | NA | 1 hypotension | NA | NA |
| Buckley et al (2016)^19^ | Cost | NA | 38 (out of 80 ADT group) in 90 days | NA | NA | NA | NA | NA |
| Buckley et al (2016)^35^ | Weight loss | Re-hospitalization | 18% 30 days  22% 60 days | NA | 2% 60 days | None | NA | NA |
| Al-Ani et al (2019)^36^ | NA | NA | 37% 30 days | NA | NA | hypotension (n=1), hypokalaemia (n=1) | NA | NA |
| Buckley et al (2019)^20^ | HF hospitalizations after 90 days | Cost of treatment | ambulatory- 38  standard - 15  double check | NA | NA | NA | NA | NA |
| Gilotra et al (2018)^37^ | Urine output | Weight loss | 52% SC 42% IV 30 days | NA | NA | 1 hypokalaemia | NA | NA |
| Sica et al (2018)^38^ | Study 1 - to assess the pharmacokinetic profile of a novel formulation of SC furosemide and record the resulting diuresis and natriuresis.  Study 2 - estimate the bioavailability of SC furosemide compared with an  equivalent dose of oral or IV furosemide in patients with  with chronic stable HF. | Assessment of local tolerance and pharmacodynamic assessment. | NA | NA | NA | (n=9) – mild erythema  (n=1) – well-defined erythema  (n=6) - swelling | NA | NA |
| Zuzarte et al (2018)^21^ | NA | NA | 0 | 5 patients within 30 days. | 3% (4/138) during 12-month follow-up. 1 patient (0.7%)  had a cardiovascular related cause of death. | No significant adverse  effects were noted | Infused group:  NT-proBNP levels were 3772 ng/L at baseline. At 3 months:  decrease to 2056 ng/L (p<0.05). | NA |
| Greene et al (2019)^26^ | NA | HF 30-day risk-standardized all-cause readmission rate (RSRR) and risk-standardized all-cause mortality rate (RSMR) | NA | Outpatient IV diuretic use was  not associated with lower absolute 30-day RSRR (−0.10% [standard error 0.06, p=0.11]) after adjustment. | Outpatient IV diuretic use was  not associated with lower absolute 30-day RSMR (−0.10% [standard error 0.06, p=0.10]). | NA | NA | NA |
| St Amand et al (2020)^39^ | time to all-cause rehospitalization or death | time to heart failure rehospitalization or death | NA | NA | 2 (7.4%)  during 180 days of follow-up (p=0.85). | NA | NA | NA |
| Ioannou et al (2020)^22^ | emergency admissions | cost of treatment | NA | 13.9% (34 patients) n 30 days | 3.3% 60 days | NA | pre pro-BNP 5138.8 ± 7480.5 | NA |
| Alghalayini et al (2020)^23^ | Hospital admission | 30-day readmission; weight loss | NA | 13.33% 30 days | NA | NA | Post BNP 1452 | NA |
| Hamo et al (2021)^15^ | 30-day re-hospitalization for ADHF. (acute decompensated heart failure) | Beyond 30 days rates of hospitalization for all cardiac causes, cardiovascular death or myocardial infarction, all cause-death; and change in KCCQ and PHQ-9 scores. | 14 patients (15%) for ADHF at 30 days. | 23% at 30 days | 0 non/cardiac at 30 days  (beyond 30 days 16 pts -17.7%) | NA | NA | NA |
| Verma et al (2021)^40^ | HF admissions | weight loss | NA | 56% (median of 22 days from clinic visit) | 14.8% (median 171 days) | 1 severe hypokalaemia | pre-BNP 597 | NA |
| Vaishnav et al (2021)^41^ | Assess HF stability, titrate GDMT, decide need for IV diuretics.  safety outcomes - adverse effects. | Education, cardiac testing, consult with other healthcare professionals | NA | NA | 9.09% 180 days | 2 mild AKI.  Severe hypokalaemia in 2 patients - 1 high urine output and 1 severe hypokalaemia on 2 occasions (no arrhythmia or hospitalization needed) | NA | NA |
| Ahmed et al (2021)^11^ | Feasibility, safety, and efficacy of outpatient  IV diuretic treatment for the management of decompensated  HF | To analyze healthcare utilization for patients  enrolled in the HeartFailure@Home service compared with patients (controls) who received  inpatient IV diuretics (usual care). | 14.9% 30 days | 11.4% | 3.51% 30 days | 1 AKI | NA | NA |
| Salmon et al (2021)^12^ | 30-day hospitalization | 30-day mortality | NA | 17.19% at 30 days | 10.96% at 30 days | NA | NA | NA |
| Wong et al (2021)^14^ | Safety outcome: all-cause mortality within the index episode.  Clinical effectiveness outcome: number of full days alive and out of hospital within 30 days after randomization. | HF rehospitalization, all-cause death, cardiovascular death within 60 days of randomization, symptom resolution/oedema  reduction/achievement of “dry weight”. Cost – effectiveness.  Patient-centred; patient and carer satisfaction,  Quality of life assessment. | 15.4% OP vs 9.1% IP | 30.8% OP vs 18.2% IP | 7.69% at 30 days | 46% (6/13) OPM had SAE, delayed discharge,  readmission (for any reason) or death, compared to 45% IPM (5/11). | pre-BNP:  IP - 357 [251, 470],  OP - 360 [264, 699] | NA |
| Butler et al (2021)^42^ | To evaluate treatment patterns and clinical outcomes in patients under 65-year-old | NA | 12% of patients in 30 days,  23% of patients in 90 days, and 45% of patients in 365 days of a WHFE | 16% of patients in 30 days,  30% of patients in 90 days, and 56% of patients in 365 days of a WHFE | NA | NA | NA | NA |
| Fort et al (2021)^16^ | Efficacy endpoint - hospitalization at 30 days due to failure of ambulatory IV treatment. Safety endpoint - severe dyselectrolytemia that required IV treatment, symptomatic hypotension, or acute renal failure. | Cost effectiveness | NA | 17% at 30 days and 20% at 60 days (of 192 episodes)? | 4% at 30-day (40% due to HF), 14% at 6-month follow-up (53% due to HF) and 26% at 1 year (38% due to HF). | 4% (7 episodes) due to dyselectrolytemia, < 1%  (1 episode) due to symptomatic hypotension. | NT-proBNP (pre 3470 [1804–6869] vs post 3154 pg/mL [1620–4992], P = 0.013) recorded in 21 episodes only. | NA |
| Thomas et al (2021)^13^ | NA | NA | NA | IP - 6 (20.7%).  not admitted - 26 (15.1%) in 30 days | During admission: IP - 7 (19.4%). not admitted - 0.  Death at 30 days: IP - 8 (22.2%). not admitted - 5 (2.9%).  Death at 1 year: IP - 14 (38.9%). not admitted - 31 (18%) | NA | NA | NA |
| Shah et al (2016)^43^ | NA | NA | 1 | NA | NA | NA | NA | Average change in creatinine - increase of 4.87% |

**Supplementary Table 3: Quality assessments using objective risk of bias tools**

**Supplementary Table 3A: A Quality assessment of Randomized control trials reviewed using the RoB2 tool**

| **Author** | **Randomization** | **Deviations from the intended interventions** | **Missing outcome data** | **Measurement of outcome data** | **Selection of reported result** | **Overall risk of bias** |
| --- | --- | --- | --- | --- | --- | --- |
| Gilotra et al (2018)^37^ | RoB Low | RoB Low | RoB Low | RoB Low | RoB Low | RoB Low |
| Hamo et al (2021)^15^ | RoB Low | RoB Low | RoB Low | RoB Low | RoB Low | RoB Low |
| Wong et al (2021)^14^ | RoB Low | RoB Low | RoB Low | RoB Low | RoB Low | RoB Low |

RoB= Risk of Bias

**Supplementary Table 3B: A Quality assessment of the Observational studies reviewed using QUIP’s tool**

| **Author** | **Study Participation** | **Study Attrition** | **Prognostic Factor Measurement** | **Outcome Measurement** | **Study Confounding** | **Statistical Analysis and Reporting** |
| --- | --- | --- | --- | --- | --- | --- |
| Ryder et al (2008)^28^ | RoB Moderate, exclusion criteria not clear, sampling frame not clear | RoB Low | RoB Low | RoB Moderate to Low, unclear follow up period | NA | RoB Low |
| Freimark et al (2009) ^29^ | RoB Low | RoB Moderate, 83% were followed up for more than 1 year, loss of follow up was not discussed in anymore detail | RoB Moderate, doses of IV diuretic treatment not given | RoB Low | NA | RoB Low |
| Herbert et al (2011)^30^ | RoB Moderate,  exclusion criteria not clear | RoB Low | RoB Moderate,  treatment dose not clear and not fixed only 130/577 patients received IV diuretics | RoB Moderate, Length of follow up not clear | NA | RoB Low |
| Lazkani et al (2012)^31^ | RoB: Moderate, inadequate recruitment, with unclear inclusion/exclusion criteria | RoB Low | RoB Moderate, doses for treatment not stated | RoB Low | NA | RoB Low |
| Banerjee et al (2012)^17^ | RoB Low | RoB Low | RoB: Moderate Not a fixed dose or course duration | RoB Moderate, follow up period not stated, main outcomes not given initially | NA | RoB Moderate, not clearly described |
| Schipper et al (2012)^32^ | RoB High- no inclusion/ exclusion criteria | RoB High, duration of follow up not stated | RoB Low | RoB Moderate, endpoints not clear | NA | RoB High, statistical analysis not described |
| Zatarain et al (2013)^33^ | RoB Moderate, unclear inclusion criteria | RoB Low | RoB Low | RoB Low | NA | RoB Low |
| Evaluation of IV diuretics for the British Heart Foundation [Brightpurpose (2014)]^18^ | RoB Low | RoB Low | RoB Low | RoB Low | RoB Low | RoB Low |
| Makadia et al (2015)^34^ | RoB Low | RoB Low | RoB Low | RoB Low | NA | RoB Low |
| Buckley et al (2016)^19^ | RoB Low | RoB Low | RoB Low | RoB Low | NA | RoB Moderate, Vague description statistical models used |
| Buckley et al (2016)^35^ | RoB Low | RoB Low | RoB Low | RoB Low | NA | RoB Moderate, statistical models used are unclear |
| Al-Ani et al (2019)^36^ | RoB: Moderate to Low  Exclusion criteria not clear | RoB: Low | RoB: Low | RoB: Low | NA | RoB: Low |
| Buckley et al (2019)^20^ | RoB Moderate,  patient baseline characteristics not stated | RoB Low | RoB Moderate to Low, doses for IV diuretic not stated | RoB Low,  No mention of index episode mortality | NA | RoB Low |
| Sica et al (2018)^38^ | RoB Low | RoB Low | RoB Low | RoB Low | NA | RoB Low |
| Zuzarte et al (2018)^21^ | RoB Low | RoB Moderate, patients lost to follow up | RoB Moderate, dose not stated | RoB Low | NA | RoB Low |
| Greene et al (2019)^26^ | RoB Moderate, no patient characteristics | NA | RoB Moderate, no dose given | RoB Low | NA | RoB, statistical analysis not described |
| St Amand et al (2020)^39^ | RoB Low | RoB Low | RoB Moderate, no dose stated | RoB Low | NA | RoB Low |
| Ioannou et al (2020)^22^ | RoB moderate, no exclusion criteria given | RoB Low | RoB Low | RoB Low | NA | RoB Low |
| Alghalayini et al (2020)^23^ | RoB Moderate, limited baseline characteristics | RoB Low | RoB Low | RoB Low | NA | RoB Low |
| Verma et al (2021)^40^ | RoB Low | RoB Low | RoB Low | RoB Low | NA | RoB Low |
| Vaishnav et al (2021)^41^ | RoB Moderate, inclusion/exclusion criteria not clear | RoB Low | RoB Low | RoB Moderate, outcomes not definitive | NA | RoB Low |
| Ahmed et al (2021)^11^ | RoB Low | RoB Low | RoB Low | RoB Low | RoB moderate - impact of confounding variables unclear | RoB Low |
| Salmon et al (2021)^12^ | RoB Low | RoB Low | RoB Moderate, no dose stated | RoB Low | NA | RoB Low |
| Butler et al (2021)^42^ | RoB Low | NA | RoB Low | RoB Low | NA | RoB Low |
| Fort et al (2021)^16^ | RoB Low | RoB Low | RoB Low | RoB Low | NA | RoB Low |
| Thomas et al (2021)^13^ | RoB Moderate, limited baseline characteristics, inclusion/exclusion criteria not clear | RoB Low | RoB Moderate, IV treatment and doses not stated | RoB Low | RoB moderate - definition and impact of confounding variables unclear | RoB Moderate, statistical analysis not described |
| Shah et al (2016)^43^ | RoB Moderate, limited baseline characteristics, inclusion/exclusion criteria not clear | RoB Low | RoB Moderate, treatment for majority of patients given only | RoB Low | NA | RoB Moderate, statistical analysis not described |

RoB= Risk of Bias; IV= intravenous; NA= Not Applicable.

The risk of bias data provided for Salmon et al was requested from one of the authors as there was insufficient data in the abstract for a complete bias assessment. Other abstracts such as Schipper and Nyjo were given high risk of bias due to insufficient data in the abstract.

**Supplementary References**

28. Ryder M, Murphy NF, McCaffrey D, O'Loughlin C, Ledwidge M, McDonald K. Outpatient intravenous diuretic therapy; potential for marked reduction in hospitalizations for acute decompensated heart failure. Eur J Heart Fail. 2008;10(3):267-72.

29. Freimark D, Arad M, Matetzky S, DeNeen I, Gershovitz L, Morag NK, Hochberg N, Makmal Y, Shechter M. An advanced chronic heart failure day care service: a 5 year single-centre experience. Isr Med Assoc J. 2009;11(7):419-25.

30. Hebert K, Dias A, Franco E, Tamariz L, Steen D, Arcement LM. Open access to an outpatient intravenous diuresis program in a systolic heart failure disease management program. Congest Heart Fail. 2011;17(6):309-13.

31. Lazkani M, Ota KS. The role of outpatient intravenous diuretic therapy in a transitional care program for patients with heart failure: a case series. J Clin Med Res. 2012;4(6):434-8.

32. Schipper J, Domingo GRR, Dickson VV, Katz SD. Implementation of Tailored Intravenous Heart Failure Therapy in a Non-Dedicated Outpatient Infusion centre. Journal of Cardiac Failure. 2012;18.

33. Zatarain-Nicolás E, López-Díaz J, de la Fuente-Galán L, García-Pardo H, Recio-Platero A, San Román-Calvar JA. Subcutaneous infusion of furosemide administered by elastomeric pumps for decompensated heart failure treatment: initial experience. Rev Esp Cardiol (Engl Ed). 2013;66(12):1002-4.

34. Makadia S, Simmons T, Augustine S, Kovell L, Harris C, Chibungu A, Parakh K. The diuresis clinic: a new paradigm for the treatment of mild decompensated heart failure. Am J Med. 2015;128(5):527-31.

35. Buckley LF, Carter DM, Matta L, Cheng JW, Stevens C, Belenkiy RM, Burpee LJ, Young MA, Weiffenbach CS, Smallwood JA, Stevenson LW, Desai AS. Intravenous Diuretic Therapy for the Management of Heart Failure and Volume Overload in a Multidisciplinary Outpatient Unit. JACC Heart Fail. 2016;4(1):1-8.

36. Al-Ani MA, Schwartz C, Winchester D, Barry J, Cerda M, Aranda Jr JM, Ahmed MM. Outpatient Intravenous Diuretic Therapy for Acute Heart Failure: A Simplified Solution to a Formidable Problem. J Card Fail. 2020;26(9):800-1.

37. Gilotra NA, Princewill O, Marino B, Okwuosa IS, Chasler J, Almansa J, Cummings A, Rhodes P, Chambers J, Cuomo K, Russell SD. Efficacy of Intravenous Furosemide Versus a Novel, pH-Neutral Furosemide Formulation Administered Subcutaneously in Outpatients With Worsening Heart Failure. JACC Heart Fail. 2018;6(1):65-70.

38. Sica DA, Muntendam P, Myers RL, ter Maaten JM, Sale ME, de Boer RA, Pitt B. Subcutaneous Furosemide in Heart Failure: Pharmacokinetic Characteristics of a Newly Buffered Solution. JACC Basic Transl Sci. 2018;3(1):25-34.

39. St Amand A, Taveira TH, Henthorne KE, Wu WC. Ambulatory Intravenous Diuretic Clinic Associated with Short-Term Risk Reduction in Mortality and Rehospitalizations in Patients Discharged with Heart Failure. R I Med J. 2020;103(9):16-21.

40. Verma V, Zhang M, Bell M, Tarolli K, Donalson E, Vaughn J, Hickey GW. Outpatient Intravenous Diuretic Clinic: An Effective Strategy for Management of Volume Overload and Reducing Immediate Hospital Admissions. J Clin Med Res. 2021;13(4):245-51.

41. Vaishnav J, Hubbard A, Chasler JE, Lepley D, Cuomo K, Riley S, Menzel K, Fajardo J, Sharma K, Judge DP, Russell SD, Gilotra NA. Management of heart failure in cardiac amyloidosis using an ambulatory diuresis clinic. Am Heart J. 2021;233:122-31.

42. Butler J, Yang M, Sawhney B, Chakladar S, Yang L, Djatche LM. Treatment patterns and clinical outcomes among patients <65 years with a worsening heart failure event. Eur J Heart Fail. 2021;23(8):1334-42.

43. Shah S, Davies A, Hall L, Lucas-Jones S, Adenwalla F, Wong A, editors. Treating HF patients in the community with IV diuretics is safe and cost-effective. Presented in British Geriatric Society Cardiovascular Meeting 2016 and RCP-Society of Physician of Wales Joint annual update 2016.
